# Environmental and Spatial Drivers of Latrine Site Selection in Dorcas Gazelle

**DOI:** 10.1002/ece3.71800

**Published:** 2025-08-25

**Authors:** Marouane Louhichi, Marie Petretto, Olfa Tabel Hmidi, Kamel Dadi, Ali Zaidi, Mohsen Jarray, Mohsen Chammem

**Affiliations:** ^1^ Laboratory of Livestock and Wildlife (LR16IRA04), Arid Regions Institute (IRA) University of Gabes Medenine Tunisia; ^2^ Consortium Animal Biodiversity of Arid Agro‐Ecosystems (CDR2024ES04), Faculty of Sciences of Gabes University of Gabes Gabes Tunisia; ^3^ Marwell Wildlife, Colden Common Winchester UK; ^4^ Faculty of Sciences of Gabes University of Gabes Gabes Tunisia; ^5^ Laboratory of Pastoral Ecosystems and Valorization of Spontaneous Plants and Associated Microorganisms (LR16IRA03), Arid Regions Institute (IRA) University of Gabes Medenine Tunisia

**Keywords:** antelope, arid ecosystem, habitat selection, localised defecation, management, national park

## Abstract

The dorcas gazelle ( *Gazella dorcas* Linnaeus, 1788), a vulnerable and cryptic species, has experienced significant population declines in recent decades. Its survival now hinges on robust conservation efforts within protected areas. However, the species' elusive nature complicates direct observation, underscoring the need for innovative and adaptive conservation approaches. In this study, we utilised latrine mapping, a cost‐effective and non‐invasive method, to evaluate the spatial dynamics and habitat preferences of dorcas gazelles in Sidi Toui National Park, Tunisia. A total of 417 active latrines were recorded across 67 surveyed quadrats, with significant clustering in specific areas indicating selective habitat use. Spatial analysis revealed autocorrelation across seven distance classes, with Moran's *I* values ranging from a significant positive value (0.326, *p* < 0.001) at the smallest distance class (0–1500 m) to a return of clustering at the broadest scale (0.176, *p* < 0.001) after a phase of non‐significant or negative autocorrelation between 2500 and 6000 m, reflecting a multi‐scale spatial structure characterised by alternating clustering and dispersion. These findings highlight a non‐random, structured spatial pattern influenced by environmental, spatial and anthropogenic factors. Multi‐model inference identified gazelle abundance, predator abundance and topography as the primary predictors of latrine site selection, with plains and hills being particularly favoured. Secondary influences included man‐made features. These findings offer actionable insights for conservation, suggesting that future management strategies should prioritise the preservation of open habitats, ensure reliable access to water sources and minimise human disturbance in critical areas to support the spatial behaviour and ecological requirements of dorcas gazelles.

## Graphical abstract

This study investigates the environmental and spatial factors influencing latrine site selection in dorcas gazelles within Sidi Toui National Park, Tunisia, using a non‐invasive latrine mapping approach. The findings reveal a structured spatial distribution, where latrines cluster in specific areas, influenced primarily by gazelle and predator abundance, topography and anthropogenic elements.

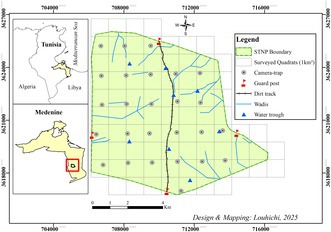

## 1 Introduction

Olfactory communication is widespread among mammals and plays a central role in territorial and social interactions (Ralls and Smith 2004; Roberts and Gosling 2004; Wronski et al. 2013; Marneweck et al. 2017a, 2017b). Among gazelle species, scent marking is particularly prominent. Males typically use pre‐orbital glands to mark individuals or objects, while ground scraping with interdigital glands is also observed (Wronski and Plath 2010). Latrines, sites of repeated defecation, carry chemical signals that provide information about the depositor, including health condition (Bowyer and Kitchen 1987), reproductive status and social rank (Miquelle 1991) and territorial boundaries (Walther et al. 1983; Wronski and Plath 2010; Attum and Mahmoud 2012). Beyond communicative, latrines may also serve hygienic purposes, reducing parasite exposure (Ezenwa 2004).

Latrine use has been documented across several antelope species, including the klipspringer ( *Oreotragus oreotragus* , Zimmermann, 1783; Roberts and Dunbar 2000), the nilgai ( *Boselaphus tragocamelus* , Pallas, 1766; Zoromski et al. 2022), the Cuvier's gazelle ( *Gazella cuvieri* , Ogilby, 1841; Gil‐Sánchez et al. 2017), the Arabian gazelle ( *Gazella arabica* , Lichtenstein, 1827; Wronski et al. 2013), the mountain gazelle ( *Gazella gazella* , Pallas, 1766; Wronski and Plath 2010), the Indian gazelle ( *Gazella bennettii* , Blyth, 1842; Schaller 1975), the goitered gazelle ( *Gazella subgutturosa* , Güldenstädt, 1780; Walther 1979), the gerenuk ( *Litocranius walleri* , Brooke, 1879; Walther 1979) and the dorcas gazelle (Attum and Mahmoud 2012), which use latrines as communication hubs for indirect social signalling (Soultan et al. 2021). Latrines serve broader ecological and social functions, enabling territory holders to advertise their presence and deterring intruders, thus reducing direct conflicts (Gosling and Roberts 2001; Rosell and Thomsen 2006; Hayward and Hayward 2010). Species‐specific differences exist like in Kirk's dik‐dik ( *Madoqua kirkii* (Günther, 1880)); both sexes mark territories through latrines (Hendrichs and Hendrichs 1971), whereas, in Arabian gazelles ( *G. arabica* ), males primarily use latrines for territorial defence and females for intraspecific communication (Wronski et al. 2013).

The non‐random placement and maintenance of latrines support their communicative function. Latrines are often located near elevated or visually prominent features to maximise detectability (Attum et al. 2006; King and Gurnell 2007; Hayward and Hayward 2010). However, in more variable or disturbed habitats, gazelles modify their strategies. In protected areas, gazelles demonstrate strong site fidelity (Baamrane et al. 2013; Abáigar et al. 2013), while in disturbed or unpredictable environments, they adopt more flexible patterns, selecting less conspicuous sites to avoid hunting or predation risks (Attum et al. 2014; Soultan et al. 2021). Thus, latrine site selection may be sensitive not only to environmental structure but also to anthropogenic pressures, an aspect that remains poorly understood in many species.

The dorcas gazelle, native to peri‐Saharan North Africa (Mallon and Kingswood 2001), is currently classified as Vulnerable on the IUCN Red List (IUCN 2017). In Tunisia, dorcas gazelles occupy a variety of arid habitats, including steppes, rocky hills and foothills (Kacem et al. 1994). Their social organisation, based on small groups and loose aggregations (Attum and Mahmoud 2012), is increasingly disrupted by habitat loss, overgrazing, illegal hunting, infrastructure development and motorised disturbance (Mallon and Kingswood 2001; Chammem et al. 2008; Baamrane et al. 2013). Given the difficulty of direct observation due to their elusive behaviour, non‐invasive methods like latrine surveys offer valuable insights into their spatial ecology, population distribution and habitat preferences (Attum et al. 2006; Bowkett et al. 2006; Wronski and Plath 2010).

Despite their importance, patterns of latrine site selection by dorcas gazelles in Tunisia remain poorly documented. Conservation and reintroduction programmes increasingly rely on latrine distribution as a proxy for habitat selection and social organisation (Hanane and Amhaouch 2021). Yet, it remains unclear whether latrine placement reflects overall habitat preferences or targeted behavioural strategies such as predator avoidance. This study aims to identify the environmental, spatial and anthropogenic drivers of latrine site selection in dorcas gazelles within Sidi Toui National Park (STNP), Tunisia. We specifically examine the roles of topography, vegetation structure, predator presence, particularly the African golden wolf ( *Canis aureus* Linnaeus, 1758) and human disturbance (e.g., proximity to fences and guard posts). We hypothesise that dorcas gazelles favour elevated, open areas that enhance visibility and facilitate social communication, while avoiding locations associated with high predator or anthropogenic risks. A clearer understanding of these spatial drivers will contribute to evidence‐based conservation planning and more effective habitat management in arid ecosystems.

## 2 Materials and Methods

### 2.1 Study Area

**FIGURE 1:**
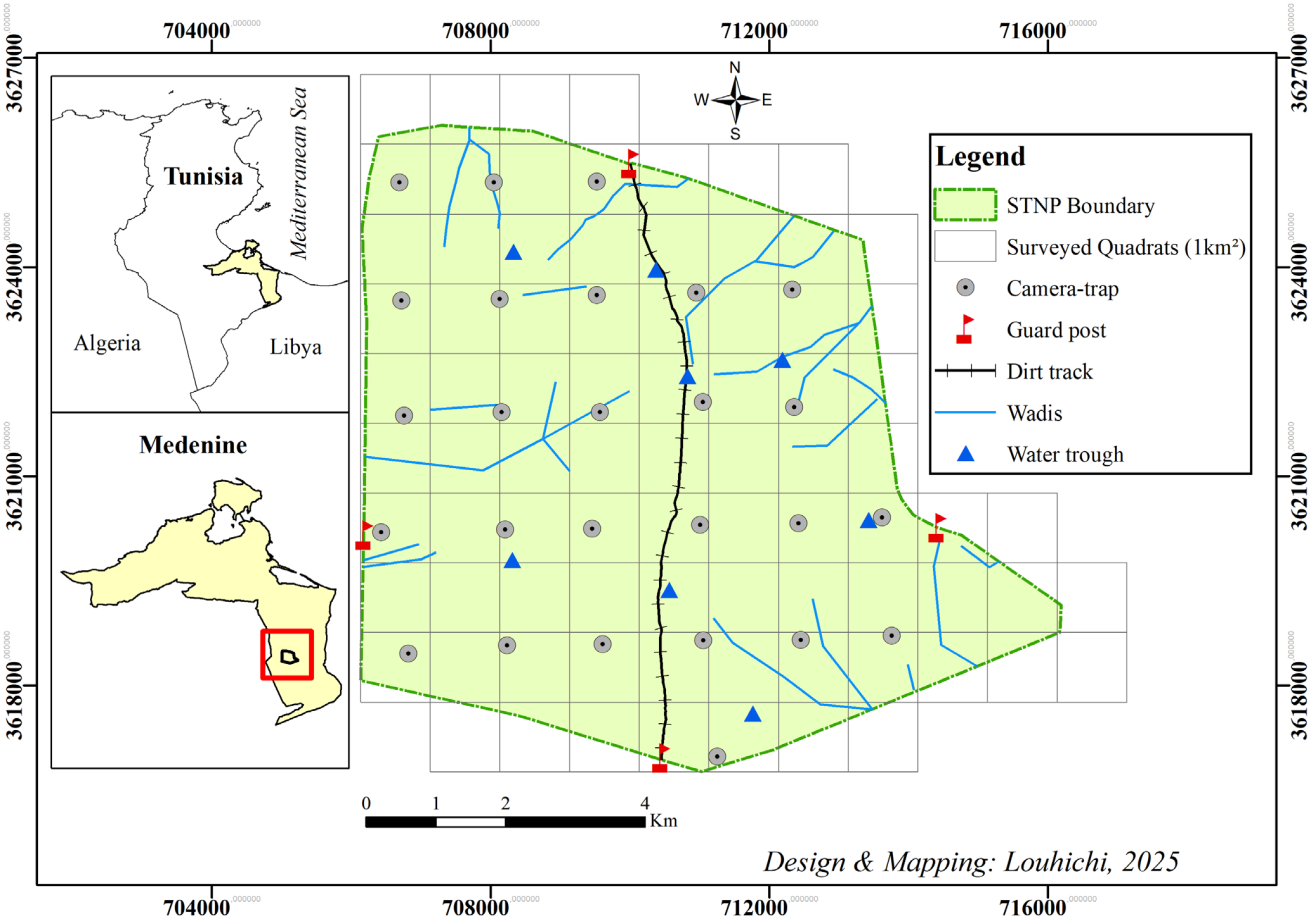
Map of STNP showing location, boundaries, infrastructure and sampling quadrats.

Data were collected throughout the STNP, located in southeastern Tunisia on the edge of the Sahara Desert near the Tunisian–Libyan border, approximately 54 km south of Ben Gardane in the Medenine Governorate (11.24° E, 32.70° N) (Figure 1). The park spans a fenced area of 63.15 km^2^ and reaches a maximum elevation of 172 m above sea level, lying at the intersection of the upper Saharan temperate zone and the lower arid cool zone. This region experiences low and irregular rainfall, with annual precipitation ranging from 100 to 125 mm. Summers are extremely hot and dry, with temperatures often reaching 45°C (Louhichi et al. 2024).

The vegetation of STNP is dominated by chamaephyte species, including *Anthyllis henoniana* Coss. ex Batt., *Gymnocarpos decander* Forssk., *Helianthemum kahiricum* Delile, *Periploca angustifolia* Labill., *Rhanterium suaveolens* Desf., *Stipagrostis pungens (Desf.) De Winter, Stipa lagascae* Roem. & Schult. and *Ziziphus lotus* (L.) Lam. (Tarhouni et al. 2014). The park supports a diverse range of fauna, including the African golden wolf, red fox ( *Vulpes vulpes* Linnaeus, 1758), Cape hare ( *Lepus capensis* Linnaeus, 1758), crested porcupine ( *Hystrix cristata* Linnaeus, 1758) and Sahelo‐Saharan species such as the scimitar‐horned oryx ( *Oryx dammah* Cretzschmar, 1826) and dorcas gazelle, along with the North African ostrich ( *Struthio camelus camelus* Linnaeus, 1758).

### 2.2 Latrine Survey

Field investigations were conducted in STNP over three months (March, April and May 2021) by three trained investigators walking parallel transects spaced 25 m apart, chosen as a compromise between detection efficiency and logistical feasibility. All surveys were conducted during the early morning (06:00–10:00) and late afternoon (16:00–18:00) hours (±1 h depending on sunrise and sunset). These time periods were selected to coincide with peak gazelle activity during this season (Abáigar et al. 2018; Louhichi et al. 2024), to minimise disturbance during the hottest and least active hours of the day and to optimise latrine detection under favourable lighting conditions. The relatively open habitat, characterised by sparse vegetation and few visual obstacles, enabled consistent detection across habitat types. Although the effective detection distance was approximately 8–10 m on either side of the transect line, the overlap between adjacent transects ensured sufficient ground coverage. To cover a 1 km^2^ area, a total of 40 parallel transects were established, allowing systematic sampling while minimising double‐counting (Wronski et al. 2013). Latrines were defined as sites of faecal accumulation with a diameter of at least 0.5 m, each containing a minimum of two distinct faecal pellet groups (Wronski and Plath 2010). Surveys encompassed various habitats, including plains, hills, mountains and wadis, ensuring comprehensive spatial representation. Each active latrine encountered was geolocated using a GPS device (Garmin 60Cx), and the coordinates were later converted into UTM format for spatial analysis using GIS tools. The park was divided into 67 quadrats, each measuring 1 km^2^, to record latrine counts and associate each quadrat with specific habitat characteristics (Figure 1).

### 2.3 Predictor Variables Abundance, Ecological and Environmental Factors

We characterised a total of 23 predictor variables within each 1 km^2^ quadrat based on their distinct properties, grouping them into three categories (Table 1): (i) Species abundance (gazelles and predators), (ii) environmental and habitat variables and (iii) anthropogenic variables.

**TABLE 1:** Summary of ecological, environmental and human disturbance factors influencing the placement of dorcas gazelle latrines.

Category	Description	Variable
Species abundance	Relative abundance for	Dorcas Gazelles (AG) African golden wolf predators (AP)
Environmental and habitat variables	Vegetation groups derived from the phytosociological map by Tarhouni (2008)	*Anthyllis sericea* and *Gymnocarpos decander* (AsGd), *Cenchrus ciliaris* (Cc), *Hammada schmittiana* and *Hammada scoparia* (HsHs), *Helianthemum kahiricum* and *Anthyllis sericea* (HkAs), *Periploca angustifolia* and *Ziziphus lotus* (PaZl), *Retama raetam* and *Stipagrostis pungens* (RrSp), *Rhanterium suaveolens* and *Helianthemum kahiricum* (RsHk), *Stipa lagascae* (Sl), *Stipagrostis pungens* (Sp).
	Substrate type	Clay (Cl), Rocky (Ro), Sandy (Sa).
	Topography	Plain (PL: 76–120 m), Hill (HI: 121–150 m) Mountain (MO: 151–172 m), Length of wadis (WA).
Anthropogenic variables	Distances from the centre of each quadrat to the nearest	Artificial water trough (WT), Dirt track (TR), Fence (FE), Guard post (GP), Shade structure (SS).

Species abundance was assessed using data from 26 camera traps (Bushnell Trophy Cam HD Aggressor; Bushnell Outdoor Products, Overland Park, KS, USA) deployed across the park as part of an ecological monitoring programme led by Marwell Wildlife (Figure 1). Cameras were distributed based on a regular grid design with approximately 1.5 km spacing, which was appropriate given the estimated home range size of dorcas gazelles in similar environments, which ranged from 15.3 to 20.6 km^2^ based on Minimum Convex Polygon estimates for individuals monitored in the Jbil National Park (Meliane et al. 2023). Our 1.5 km grid spacing provided sufficient coverage to detect individuals within their typical movement ranges. Cameras were installed at heights of 40–50 cm and oriented towards trails or open areas to detect medium‐ to large‐bodied animals weighing ranging from over 1 kg to approximately 150 kg, typically species with shoulder heights compatible with this setup above 30–50 cm, such as dorcas gazelles, African golden wolves and scimitar‐horned oryx (Kacem et al. 1994; Taha et al. 2018; Meliane et al. 2023). Cameras operated continuously (24 h/day) and captured a series of three high‐resolution images per trigger. Detection events were consolidated manually, and independent events were defined as photographs of the same species separated by ≥ 30 min (Jiménez et al. 2017; Agha et al. 2018). Predation was considered through the presence of the African golden wolf, the main predator of the dorcas gazelle in the park and the largest predator in southern Tunisia (Karssene et al. 2019). Interspecific competition was not explicitly included as a predictor variable, as previous research indicated clear spatial segregation between dorcas gazelles and other sympatric herbivores, notably the scimitar‐horned oryx (Louhichi et al. 2024).

The relative abundance index (RAI) for dorcas gazelles (AG) and African golden wolves (AP) was calculated for each camera site (*i*) following the formula (Louhichi et al. 2024):

RAIi=IDi×100/TDi

where ID*ᵢ* is the number of independent detections of each species and TD*ᵢ* = total number of trap‐days for camera *i*.

The ecological and environmental habitat variables considered relevant to wildlife presence and behaviour, including vegetation types, substrate type, topography and levels of human disturbance (Bergeson et al. 2013), are compiled in Table 1.

### 2.4 Data Analysis

#### 2.4.1 Spatial Data Processing

We used ArcGIS (Version 10.8, Environmental Systems Research Institute Inc., Redlands, CA, USA, 2023) to divide the park map into 1 km^2^ quadrats, map GPS coordinates of latrine locations, and perform Kernel Density Estimation (KDE) at 95% and 50% thresholds to assess their spatial distribution and identify areas of higher density. For each quadrat, we extracted KDE values by overlaying the density surfaces, and in cases where multiple KDE contours overlapped within the same quadrat, we assigned the highest KDE value observed to represent that quadrat.

Additionally, ArcGIS was used to calculate all environmental and spatial variables associated with each quadrat (e.g., vegetation areas, length of wadis, anthropogenic features). Additionally, ArcGIS was used to calculate all environmental and spatial variables associated with each quadrat (e.g., vegetation areas, length of wadis, anthropogenic features). This software was also employed to classify habitat types (plains, hills and mountains) based on a digital elevation model (DEM) of the study area above sea level (Table 1), ensuring a consistent and ecologically meaningful categorisation of topographic features. This approach enabled a detailed spatial analysis of latrine distribution and provided insights into habitat selection and territorial marking of dorcas gazelles (McGarigal et al. 2016). Camera trap locations and quadrat boundaries were mapped and visualised using QGIS (QGIS Geographic Information System, 2018).

#### 2.4.2 Spatial Autocorrelation

To evaluate spatial patterns in the distribution of gazelle latrines, we calculated Moran's *I* (Moran 1950), a widely used index for quantifying spatial autocorrelation in ecological data (Dormann et al. 2007). Moran's *I* measures the degree to which the presence of latrines in a given quadrat is correlated with their presence in neighbouring quadrats. Values range from −1 to +1, with positive values indicating clustering, negative values suggesting dispersion and values near zero reflecting randomness (Fortin and Dale 2005). This analysis was also applied to explanatory variables that positively influenced gazelle latrine sites selection, using version 4.0 of the SAM programme (Spatial Analysis in Macroecology; Rangel et al. 2010). To define distance classes for the analysis, we applied Sturges' rule (Sturges 1926), commonly used in ecological and statistical studies to estimate optimal interval numbers for continuous data. Based on the 67 quadrats, the formula *K* = 1 + 3.322×log_10_ (*N*), yielded approximately seven distance classes. These were used to partition the full range of pairwise distances between quadrats into the following intervals: 0–1500 m, 1501–2500 m, 2501–3500 m, 3501–4236 m, 4237–4929 m, 4930–6047 m and 6048–8762 m. Additionally, we applied the Bonferroni correction to assess the significance of values in the correlogram, an essential approach step to limit the risk of type I error due to multiple comparisons (Armstrong 2014). This method adjusts the α threshold by dividing it by the number of comparisons, ensuring a more rigorous interpretation of significance. With seven distance classes, the corrected significance level was set at 0.01 (0.05/7 = 0.007). Following (Legendre and Fortin 1989), a correlogram was considered significant if it included at least one Moran's *I* value exceeding this α’ threshold.

#### 2.4.3 Modelling Habitat Selection

To conduct this part of the modelling, we used RStudio (R Core Team 2023, Version 4.3.2) along with the “MuMIn” package (Version 1.47.5) for multi‐model inference (MMI). This approach complements Moran's *I* by integrating additional spatial analysis methods that provide a more nuanced understanding of spatial patterns, especially when incorporating ecological predictors to explain spatial distributions and uncover underlying processes. Specifically, we employed Generalised Linear Models (GLMs) in combination with information‐theoretic methods, MMI and adjustments for spatial autocorrelation and multicollinearity. These methods effectively address several criticisms commonly associated with spatial analyses based solely on Moran's *I*. Consequently, we applied MMI techniques to spatial data on the number of gazelle latrines per quadrat to identify the key factors influencing habitat selection (Anderson and Burnham 2004).

To examine the relationship between gazelle latrines and the explanatory variables, we used GLMs with a Poisson distribution (Guisan et al. 2002). To address issues of autocorrelation and multicollinearity, we applied Spearman's rho correlation coefficient to assess pairwise correlations among the predictor variables and calculated the variance inflation factor (VIF). We retained predictors with correlation coefficients < 0.8 and VIF values < 10 (Pradhan 2016).

We used the Akaike Information Criterion (AIC) (McCullagh 2019) to compare alternative models and employed AICc to account for small sample sizes (*n*/*k* < 40: *n* quadrats = 67 and *k* variables = 14) (Anderson and Burnham 2004). For each model in the dataset, we calculated AICc, considering the model with the lowest AICc value (AICcmin) as the best and most parsimonious fit. Among competing models, we used MMI to identify the best fit (Anderson and Burnham 2004), ranking candidate models by calculating the AICc differences (Δi) relative to AICcmin. A larger Δi indicates a weaker model, while models with Δi < 2 were considered not significantly different (Anderson and Burnham 2004).

## 3 Result

We evaluated the relative importance of predictors in determining habitat selection using two approaches: (1) Predictor selection probability (SP), representing the likelihood of a predictor's inclusion in the top models if the analysis were repeated on a different dataset (Whittingham et al. 2006); and (2) Model‐averaged coefficients, which reflect each predictor's contribution to variation in the habitat selection index. Additionally, we assessed the agreement between the best model and explanatory variables using adjusted *R* ^2^ , where *R* ^2^ > 0.40 indicated a robust model with strong predictive capabilities.

### 3.1 Spatial Patterns, Autocorrelation and Site‐Use of Gazelle Latrines

**FIGURE 2:**
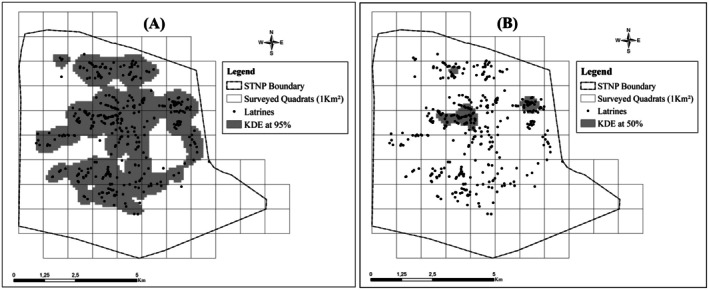
Map of latrine distribution in STNP: (A) KDE at 95% and (B) KDE at 50%.

We recorded a total of 417 gazelle latrines in 42 of the 67 surveyed quadrats in STNP, with most latrines concentrated near the park's centre, as shown by both the 95% and 50% KDE (Figure 2A,B). The estimated surface area of the 95% KDE was approximately 23.47 km^2^ and 0.30 km^2^ for the two patches, which were spatially close, separated by about 200 m. The 50% KDE delineated three core patches, covering 0.10, 0.42 and 1.03 km^2^, respectively, with a mean distance of approximately 1900 m between them. Latrines were predominantly found in open habitats, particularly on hills (*n* = 274, 65.71%), and in rocky terrain (*n* = 206, 49.40%). Their spatial distribution was irregular, with elevated densities observed in particular areas, suggesting selective habitat use. Spatial analyses further confirmed this non‐random pattern, revealing localised clustering and scale‐dependent spatial structure. KDE highlighted several discrete patches of high latrine density, with three core areas (50% KDE) covering approximately 2.45% of the study area. These clusters, primarily located within grey‐shaded zones (Figure 2B), indicate significant spatial aggregation likely driven by ecological or environmental factors.

**FIGURE 3:**
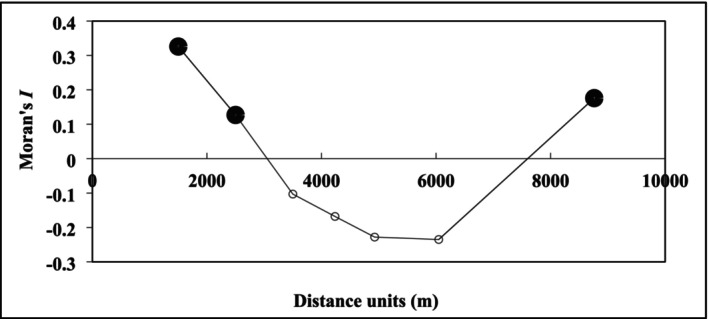
Correlogram of latrine abundance in STNP: Black circles indicate significant positive autocorrelation (Bonferroni‐corrected); Open circles indicate non‐significant autocorrelation.

Supporting this, spatial autocorrelation analysis based on Moran's *I* (Figure 3) revealed strong clustering at fine spatial scales (0–1500 m; Moran's *I* = 0.326, *p* < 0.001), which persists at intermediate distances (1501–2500 m; Moran's *I* = 0.127, *p* = 0.002), indicating persistent clustering at short to moderate ranges. Between 2500 and 6000 m, spatial structure became non‐significant or negative, indicating increasing spatial dispersion. A return to clustering was observed at broader scales (6048–8762 m; Moran's *I* = 0.176, *p* < 0.001), suggesting multi‐scale structuring of latrine use across the landscape.

Field observations further revealed that gazelles frequently revisit and maintain these sites, as fresh dung was observed in 72.18% (*n* = 301) of latrines upon subsequent visits. To complement these field observations and better understand gazelle activity, we recorded 173 independent dorcas gazelle detections over 2275 camera‐trap days, with a mean of 6.65 ± 9.12 detections per camera. In parallel, African golden wolves were detected 201 times independently, with a mean of 7.72 ± 6.18 detections per camera.

### 3.2 Habitat Selection Modelling and Spatial Structure of Environmental Predictors

#### 3.2.1 Variable Selection and MMI of Latrine Site Selection

After examining correlation coefficients and variance inflation tests (VIF), we retained thirteen predictor variables: Gazelle abundance (AG), predator abundance (AP), hills (HI), plains (PL), mountains (MO), wadis (WA), water troughs (WT), fences (FE), guard posts (GP), *Cenchrus ciliaris* (Cc), *Retama raetam* and *Stipagrostis pungens* (RrSp), *Stipa lagascae* (Sl) and *Stipagrostis pungens* (Sp). We removed the shade structure (SS) due to its strong correlation with WT, and substrate type (clay, rocky and sandy) due to its strong correlation with topography and vegetation type (*r* > 0.8). Additionally, we excluded five plant groups, AsGd, HsHs, PaZl, RsHk and HkAs, because of their high collinearity (VIF > 10) with topographic variables (mountains and plains) and other vegetation types, which could inflate variance in habitat selection estimates. Dirt tracks (TR) were also excluded, as their inclusion caused AIC computation issues in R due to the high number of variables. Moreover, this variable was not significant according to the GLM results.

The MMI approach revealed that AG, AP, HI, PL, WT and FE were the most influential predictors of latrine site selection (Table 2). The best model (AICc = 286.83, *R* ^2^ = 0.73, Wt = 0.08) included these variables along with Sl and Sp. Additional plausible models (∆AICc < 2) incorporated GP and RrSp, though with lower influence. Selection probabilities (SP) confirmed the importance of AG, FE, HI, PL and WT (SP = 0.93), followed by AP (SP = 0.91), Sl (SP = 0.87) and Sp (SP = 0.67). In contrast, GP and RrSp had lower SP values (0.42 and 0.38, respectively), suggesting a negligible influence on latrine site selection. Based on SP values and model coefficients, the predictors can be ranked in order of influence as follows: plains > hills > gazelle abundance > water troughs > fences > predator abundance > *Stipa lagascae* > *Stipagrostis pungens*.

**TABLE 2:** Information‐theoretic and multi‐model inference (MMI) results for gazelle latrine habitat selection in STNP.

Included predictors												AICc	∆AICc	Wt	R^2^
	AG	AP	Cc	FE		HI	PL		Sl	Sp	WT	**271.58**	**0.00**	**0.08**	**0.73**
	AG	AP		FE		HI	PL	RrSp	Sl	Sp	WT	272.10	0.52	0.06	
	AG	AP		FE	GP	HI	PL		Sl	Sp	WT	272.16	0.58	0.06	
	AG	AP		FE	GP	HI	PL	RrSp	Sl	Sp	WT	272.19	0.61	0.06	
	AG	AP		FE		HI	PL		Sl	Sp	WT	272.50	0.91	0.05	
	AG	AP	Cc	FE	GP	HI	PL		Sl	Sp	WT	272.90	1.32	0.04	
Selection probability (SP)	0.93	0.91	0.36	0.93	0.42	0.93	0.93	0.38	0.87	0.67	0.93				
Coefficient (e‐03)	44.730	30.500	0.001	0.276	−0.070	73.390	118.400	0.000	−0.008	0.003	−0.497				
SE (e‐03)	14.120	12.920	0.002	0.080	0.097	5.874	14.420	0.000	0.006	0.002	0.132				

*Note:* The best‐supported model is shown in bold. For each predictor in this model, AICc, ∆AICc, Akaike weight (Wt) and *R* ^2^ are reported. Selection probabilities (SP), model‐averaged coefficient and standard error (SE) are provided for all predictors. Predictors are abundance of gazelles (AG), abundance of predators (AP), fences (FE), guard posts (GP), hills (HI), plains (PL), *Cenchrus ciliaris* (Cc), *Retama raetam* and *Stipagrostis pungens* (RrSp), *Stipa lagascae* (Sl), *Stipagrostis pungens* (Sp) and water troughs (WT).

### 3.3 Spatial Autocorrelation of Habitat Selection Predictors

Spatial autocorrelation analyses revealed that several habitat selection predictors exhibited scale‐dependent spatial structures (Table 3). AG showed strong clustering in the first distance class (1500 m, Moran's *I* = 0.254, *p* < 0.001), followed by significant dispersion at the third distance class (3500 m, *I* = −0.145, *p* = 0.005) and fourth distance class (4236 m, *I* = −0.156, *p* = 0.006), and re‐clustering at the broadest scale (seventh class, 8762 m, *I* = 0.098, *p* = 0.001). Similarly, AP showed strong local clustering in the first class (1500 m, *I* = 0.181, *p* < 0.001), remained random at mid‐scales and exhibited strong dispersion in the seventh class (8762 m, *I* = −0.252, *p* < 0.001). For topographic predictors, HI displayed clustering at the first class (1500 m, *I* = 0.278, *p* < 0.001), randomness at mid‐distances, dispersed between the fifth and sixth classes (4929–6047 m, *I* = −0.217 and −0.177, *p* < 0.001) and clustering again at broad scales (8762 m, *I* = 0.126, *p* < 0.001). PL showed weak to moderate clustering at both short (1500 m) and broad (8762 m) scales, with non‐significant or weak dispersion in between.

**TABLE 3:** Spatial autocorrelation of habitat selection predictors with Moran's *I* and *p*‐values.

Classes	Class 1		Class 2		Class 3		Class 4		Class 5		Class 6		Class 7	
Distances (m)	1500		2500		3500		4236		4929		6047		8762	
Predictors	Moran's *I*	*p*	Moran's *I*	*p*	Moran's *I*	*p*	Moran's *I*	*p*	Moran's *I*	*p*	Moran's *I*	*p*	Moran's *I*	*p*
Abundance of gazelle AG)	0.254	**< 0.001**	−0.044	0.543	−0.145	**0.005**	−0.156	**0.006**	−0.074	0.223	−0.033	0.663	0.098	**0.001**
Abundance of predator (AP)	0.181	**< 0.001**	−0.038	0.632	−0.037	0.641	0.057	0.163	0.130	**0.003**	−0.140	**0.002**	−0.252	**< 0.001**
Fences (FE)	0.575	**0.000**	0.104	0.013	−0.218	**< 0.001**	−0.372	**< 0.001**	−0.404	**0.000**	−0.171	**< 0.001**	0.372	**0.000**
Hills (HI)	0.278	**< 0.001**	0.064	0.084	−0.065	0.261	−0.115	0.047	−0.217	**< 0.001**	−0.177	**< 0.001**	0.126	**< 0.001**
Plains (PL)	0.105	0.012	0.016	0.487	−0.057	0.335	−0.119	0.034	−0.063	0.301	−0.105	0.025	0.123	**< 0.001**
*Stipa lagascae* (Sl)	0.249	**< 0.001**	−0.023	0.843	−0.023	0.831	−0.031	0.718	−0.041	0.520	−0.076	0.097	−0.171	**< 0.001**
*Stipagrostis pungens* (Sp)	−0.019	0.727	−0.018	0.849	−0.020	0.760	−0.021	0.760	−0.015	0.990	−0.012	0.913	0.001	0.708
Water troughs (WT)	0.187	< 0.001	−0.049	0.476	0.034	0.289	−0.057	0.419	−0.054	0.429	−0.189	< 0.001	0.030	0.194

*Note:* Significant *p* in bold.

Regarding vegetation types, Sl clustered at 1500 m (*I* = 0.249, *p* < 0.001) but became increasingly dispersed, significantly so at 8762 m (*I* = −0.171, *p* < 0.001), whereas Sp showed no significant spatial structure across all classes. For anthropogenic features, FE demonstrated the most pronounced spatial structure, with intense clustering at 1500 m (*I* = 0.575, *p* < 0.001), significant dispersion from 3500 to 6047 m (*I* = −0.218 to −0.404, all *p* < 0.001) and re‐clustering at 8762 m (*I* = 0.372, *p* < 0.001). Lastly, water‐related infrastructure, WT was clustered at 1500 m (*I* = 0.187, *p* < 0.001) but dispersed at 6047 m (*I* = −0.189, *p* < 0.001), with no clear spatial pattern at other scales.

## 4 Discussion

Our findings highlight the complex interplay of environmental, spatial and anthropogenic factors shaping latrine site selection in dorcas gazelles within STNP. The observed structured spatial distribution of latrines is clearly non‐random, supporting our hypothesis that latrine distribution is structured by habitat characteristics, species distribution and anthropogenic factors. Among the habitat variables, plains had the strongest effect on latrine site selection, followed by hills, whereas mountains had no significant influence. This suggests a clear preference for open, accessible areas, which likely enhance predator detection and facilitate movement, consistent with previous findings showing that dorcas gazelles favour sparsely vegetated rocky plains over denser vegetation (Abáigar et al. 2013; Cooke et al. 2016; Meliane et al. 2023). Interestingly, predator presence was frequently recorded in quadrats containing predators, indicating that gazelles may tolerate some level of predator risk or that habitat overlap and resource constraints limit their ability to avoid these areas.

In addition to topography and predator presence, gazelle abundance emerged as a significant factor in latrine distribution, emphasising the role of social dynamics in site selection. This supports previous research showing that latrines are not simply the result of random defecation but serve as social communication hubs, with clustering patterns reflecting group behaviour (Wronski et al. 2013; Soultan et al. 2021). Spatial analyses further support this, with significantly positive Moran's *I* values at smaller distance classes pointing to a strong clustering of latrines. Such clustering within specific quadrats may represent critical nodes within gazelle home ranges, potentially driven by conspecific density and social interactions (Wronski and Plath 2010; Zoromski et al. 2022). As shown in Arabian gazelles and other species, higher latrine densities within home range cores are often linked to internal social communication rather than territorial marking (Wronski et al. 2013). As distance increases, this clustering gives way to significant dispersion, with negative Moran's *I* values reflecting a spatial separation likely aimed at avoiding oversaturation of latrines in heavily used areas. This broader dispersion pattern may be reinforced by resource heterogeneity linked to variable precipitation, which affects both habitat quality and gazelle distribution (Attum et al. 2014). For instance, areas recently exposed to rainfall may temporarily support higher herbivore densities, thus influencing where latrine clusters form.

Gazelle abundance emerged as one of the strongest predictors of latrine site selection, further highlighting the role of social dynamics in shaping latrine distribution. High latrine densities in areas with increased gazelle activity suggest these sites serve important functions in intra‐specific communication, similar to patterns observed in other territorial ungulates such as the mountain gazelle and nilgai (Wronski and Plath 2010; Zoromski et al. 2022). This spatial association reinforces the idea that latrines function not merely as waste sites but as markers of social interaction, dominance and territory use within gazelle populations. Predator abundance also significantly influenced latrine distribution, supporting the hypothesis that gazelles may use latrines as part of a risk assessment strategy (Navarro‐Castilla et al. 2019). The clustering of latrines in open areas, where visual detection of predators is enhanced, may represent an adaptive anti‐predator behaviour, facilitating early detection and escape, a pattern reported in various mammals (Bonenfant and Kramer 1996; Caro 2005; Navarro‐Castilla et al. 2019). Alternatively, the consistent use of latrines by both males and females, as observed in enclosure settings, suggests a potential role in reproductive communication (Abáigar et al. 2013). Moreover, anthropogenic disturbance may further shape latrine site selection. Soultan et al. (2021) found that gazelles altered their latrine placement in response to human pressure, favouring less conspicuous or more sheltered sites in highly disturbed areas. This behavioural plasticity implies that latrine use may serve multiple adaptive functions, including communication, risk management and reproductive signalling, depending on the environmental and social context.

Our results confirm that topography is a key determinant of latrine site selection, with gazelles showing a preference for both hills and plains, though for different reasons. While more latrines were recorded in hills, likely due to their larger surface area in the park, the model indicated a stronger selection for plains, as indicated by their higher coefficient and selection probability. This suggests that gazelles actively prefer plains for latrine placement when such habitats are accessible, despite the higher absolute number of latrines in hill areas. Although hills may offer elevated vantage points, it is likely the increased visibility in open habitats that enhances predator detection, a strategy observed in several ungulate species that depend on unobstructed landscapes to anticipate threats (Brashares and Arcese 2002; Pays et al. 2012). Plains may also offer easier access, more stable substrate conditions and lower energetic costs for movement, making them more favourable when available. Furthermore, as reported by Attum and Mahmoud (2012), dorcas gazelles may use trees of different sizes for feeding and social purposes, suggesting that in tree‐sparse areas, open visibility becomes even more crucial for both survival and communication.

Anthropogenic features such as fences and water troughs significantly influenced latrine distribution, though their effects were secondary to topographic and ecological factors. The positive coefficient for the distance to fences indicates that gazelles preferentially establish latrines farther from these structures, suggesting avoidance, likely driven by increased human disturbance, restricted movement, or the risk of predator entrapment near fences (Hayward and Kerley 2009; Woodroffe et al. 2014). This pattern aligns with broader trends in human‐wildlife interactions, as many species tend to avoid areas frequented by humans (Muhly et al. 2011; Tucker et al. 2018). Although features, such as fences, guard posts and water troughs, were less influential than primary factors, their role in shaping spatial behaviour remains notable. In protected environments, Vanak et al. (2010) found that fences may both protect from and restrict movement, creating areas where animals feel secure but limited, which influences latrine site distribution. Soultan et al. (2021) found that gazelles altered their latrine placement in response to human pressure, favouring more sheltered or hidden sites in highly disturbed areas. This behavioural plasticity implies that latrine use may serve multiple adaptive functions, including communication, risk management and reproductive signalling, depending on the environmental and social context. In contrast, the negative coefficient for the distance to water troughs suggests that gazelles are more likely to establish latrines closer to these locations. While gazelles do not depend physiologically on surface water, as they meet hydration needs through succulent plants (Martin 2000; Ostrowski and Williams 2006; Babor et al. 2014), water troughs may act as focal points for animal interactions and territoriality. Thus, latrine clustering near troughs likely reflects frequent use and conspecific encounters rather than water dependence alone (Owen‐Smith 1996). A similar pattern of gazelle aggregation near water sources was also observed in Jbil National Park by Meliane et al. (2023), under comparable environmental and climatic conditions.

Vegetation composition played a secondary but notable role in latrine site selection. The negative association with *Stipa lagascae* suggests that gazelles are less likely to establish latrines in areas dominated by this species, a pattern consistent with Cooke et al. (2016), who found that dorcas gazelles prefer open, sparsely vegetated plains over denser vegetation. In contrast, the positive association with *Stipagrostis pungens* suggests a preference for open grassland habitats that facilitate predator detection and social interactions (Abáigar et al. 2013). However, other vegetation types such as *Cenchrus ciliaris* had low selection probabilities, suggesting that not all grass species contribute equally to habitat selection for latrines, likely because this group is mostly located on mountain summits within the park.

Finally, our findings reinforce the value of latrine density as a non‐invasive tool for population monitoring and habitat use, as highlighted by Wronski and Plath (2010) and Wronski et al. (2013). These findings have important implications for conservation planning in arid landscapes. The dorcas gazelle's preference for open habitats and avoidance of human infrastructure should inform habitat management strategies that maintain undisturbed core areas and functional landscape connectivity. Additionally, the strategic placement of water points could help guide animal movement patterns and reduce habitat fragmentation.

We believe that this clustering pattern reflects both environmental and behavioural influences. In particular, latrine locations may be shaped by trade‐offs between territorial marking and predator avoidance, responses to human disturbance and proximity to feeding areas—factors commonly associated with gazelle behaviour.

Future research should explore seasonal variation and climatic influences, such as temperature and precipitation, which are known to affect gazelle distribution (Attum et al. 2014). Incorporating these variables into habitat suitability models could improve predictions under changing climatic conditions. While our study focused on spatial, environmental and anthropogenic predictors, we acknowledge that other variables, such as behavioural traits including dominance, mating activity or reproductive status, could also influence latrine placement, though these remain difficult to assess due to logistical constraints. Nonetheless, our interpretation of predictors as proxies for behavioural responses enhances understanding of the ecological drivers of latrine distribution.

## 5 Conclusion

Latrine site selection in dorcas gazelles is a multifaceted process shaped by biotic interactions, topography and anthropogenic influences. The observed clustering at small scales and dispersion at broader distances reflect a balance between social, ecological and safety considerations. These spatial patterns likely result from trade‐offs between territorial marking and predator avoidance, responses to human disturbance and proximity to feeding areas—factors commonly influencing gazelle behaviour. These findings underscore the importance of spatially explicit conservation strategies that align habitat management with gazelle behavioural ecology. By preserving key habitat features and mitigating anthropogenic disturbances, conservation efforts can support the long‐term persistence of dorcas gazelle populations in STNP and other arid landscapes.

## Author Contributions

**Marouane Louhichi:** conceptualization (equal), data curation (equal), formal analysis (equal), investigation (equal), methodology (equal), project administration (equal), software (equal), supervision (equal), validation (equal), visualization (equal), writing – original draft (equal), writing – review and editing (equal). **Marie Petretto:** investigation (equal), resources (equal), validation (equal), writing – review and editing (equal). **Olfa Tabel Hmidi:** formal analysis (equal), software (equal). **Kamel Dadi:** investigation (equal). **Ali Zaidi:** investigation (equal). **Mohsen Jarray:** investigation (equal). **Mohsen Chammem:** supervision (equal), validation (equal), visualization (equal), writing – review and editing (equal).

## Ethics Statement

Gazelle monitoring for the purpose of this study was achieved through motion‐sensitive cameras. No animals were caught or manipulated for the purpose of this study. Motion‐sensitive camera studies in Southern Tunisia have been subject to Marwell Wildlife's ethical review process.

## Conflicts of Interest

The authors declare no conflicts of interest.

## Supporting information


Data S1.

Data S2.

## Data Availability

The data supporting the findings of this study are included within the main document and are available upon reasonable request.

## References

[ece371800-bib-0001] Abáigar, T. , M. Cano , and C. Ensenyat . 2013. “Habitat Preference of Reintroduced Dorcas Gazelles (* Gazella dorcas Neglecta*) in North Ferlo, Senegal.” Journal of Arid Environments 97: 176–181. 10.1016/j.jaridenv.2013.06.004.

[ece371800-bib-0002] Abáigar, T. , M. Cano , and C. Ensenyat . 2018. “Time Allocation and Patterns of Activity of the Dorcas Gazelle ( *Gazella dorcas* ) in a Sahelian Habitat.” Mammal Research 63: 73–82. 10.1007/s13364-017-0334-0.

[ece371800-bib-0003] Agha, M. , T. Batter , E. C. Bolas , et al. 2018. “A Review of Wildlife Camera Trapping Trends Across Africa.” African Journal of Ecology 56, no. 4: 694–701. 10.1111/aje.12565.

[ece371800-bib-0004] Anderson, D. , and K. Burnham . 2004. Model Selection and Multi‐Model Inference. Vol. 63. Second ed, 10. NY: Springer‐Verlag.

[ece371800-bib-0005] Armstrong, R. A. 2014. “When to Use the Bonferroni Correction.” Ophthalmic and Physiological Optics 34, no. 5: 502–508. 10.1111/opo.12131. 24697967

[ece371800-bib-0006] Attum, O. , P. Eason , and S. Wakefield . 2006. “Conservation Implications of Midden Selection and Use in an Endangered Gazelle ( *Gazella gazella* ).” Journal of Zoology 268, no. 3: 255–260. 10.1111/j.1469-7998.2005.00027.x.

[ece371800-bib-0007] Attum, O. , U. Ghazali , S. K. El Noby , and I. N. Hassan . 2014. “The Effects of Precipitation History on the Kilometric Index of Dorcas Gazelles.” Journal of Arid Environments 102: 113–116. 10.1016/j.jaridenv.2013.11.009.

[ece371800-bib-0008] Attum, O. , and T. Mahmoud . 2012. “Dorcas Gazelle and Livestock Use of Trees According to Size in a Hyper‐Arid Landscape.” Journal of Arid Environments 76: 49–53. 10.1016/j.jaridenv.2011.07.002.

[ece371800-bib-0009] Baamrane, M. A. A. , M. Znari , C. Loggers , S. El Mercht , and M. Naimi . 2013. “Demographic Decline of the Last Surviving Moroccan Dorcas Gazelles *Gazella Dorcas Massaesyla* in M'Sabih Talaa Reserve, Morocco.” Oryx 47, no. 4: 578–583. 10.1017/S0030605312000506.

[ece371800-bib-0010] Babor, H. , A. B. Okab , E. M. Samara , K. A. Abdoun , O. Al‐Tayib , and A. A. Al‐Haidary . 2014. “Adaptive Thermophysiological Adjustments of Gazelles to Survive Hot Summer Conditions.” Pakistan Journal of Zoology 46, no. 1: 245–252.

[ece371800-bib-0011] Bergeson, S. M. , T. C. Carter , and M. D. Whitby . 2013. “Partitioning of Foraging Resources Between Sympatric Indiana and Little Brown Bats.” Journal of Mammalogy 94, no. 6: 1311–1320. 10.1644/12-MAMM-A-311.

[ece371800-bib-0012] Bonenfant, M. , and D. L. Kramer . 1996. “The Influence of Distance to Burrow on Flight Initiation Distance in the Woodchuck, *Marmota Monax* .” Behavioral Ecology 7, no. 3: 299–303. 10.1093/beheco/7.3.299.

[ece371800-bib-0013] Bowkett, A. E. , N. Lunt , F. Rovero , and A. B. Plowman . 2006. “How Do You Monitor Rare and Elusive Mammals?” In Animals, Zoos and Conservation, edited by E. Zgrabczyskaňska , P. Ćwiertnia , and J. Ziomek , 21–28. Zoological Garden in Poznan.

[ece371800-bib-0014] Bowyer, R. T. , and D. W. Kitchen . 1987. “Significance of Scent‐Marking by Roosevelt Elk.” Journal of Mammalogy 68, no. 2: 418–423. https://www.jstor.org/stable/1381489.

[ece371800-bib-0015] Brashares, J. S. , and P. Arcese . 2002. “Role of Forage, Habitat and Predation in the Behavioural Plasticity of a Small African Antelope.” Journal of Animal Ecology 71, no. 4: 626–638. https://www.jstor.org/stable/1555812.

[ece371800-bib-0016] Caro, T. 2005. Antipredator Defenses in Birds and Mammals, 265–304. Univ. Chicago Press.

[ece371800-bib-0017] Chammem, M. , S. Selmi , S. Nouira , and T. Khorchani . 2008. “Factors Affecting the Distribution of Dorcas Gazelle.” Journal of Zoology 275, no. 2: 146–152. 10.1111/j.1469-7998.2008.00421.x.

[ece371800-bib-0018] Cooke, R. S. , T. Woodfine , M. Petretto , and T. H. Ezard . 2016. “Resource Partitioning Between Ungulate Populations in Arid Environments.” Ecology and Evolution 6, no. 17: 6354–6365. 10.1002/ece3.2218. 27656279 PMC5016655

[ece371800-bib-0019] Dormann, C. F. , J. M. McPherson , M. B. Araújo , et al. 2007. “Methods to Account for Spatial Autocorrelation in the Analysis of Species Distributional Data: A Review.” Ecography 30, no. 5: 609–628. 10.1111/j.2007.0906-7590.05171.x.

[ece371800-bib-0020] Ezenwa, V. O. 2004. “Selective Defecation and Selective Foraging: Antiparasite Behavior in Wild Ungulates?” Ethology 110, no. 11: 851–862. 10.1111/j.1439-0310.2004.01013.x.

[ece371800-bib-0021] Fortin, M. J. , and M. R. T. Dale . 2005. Spatial Analysis: A Guide for Ecologists. Cambridge University Press.

[ece371800-bib-0022] Gil‐Sánchez, J. M. , F. J. Herrera‐Sánchez , B. Álvarez , et al. 2017. “Evaluating Methods for Surveying the Endangered Cuvier's Gazelle *Gazella Cuvieri* in Arid Landscapes.” Oryx 51, no. 4: 648–655. 10.1017/S0030605316000430.

[ece371800-bib-0023] Gosling, L. M. , and S. C. Roberts . 2001. “Scent‐Marking by Male Mammals: Cheat‐Proof Signals to Competitors and Mates.” In Advances in the Study of Behavior, vol. 30, 169–217. Academic Press. 10.1016/S0065-3454(01)80007-3.

[ece371800-bib-0024] Guisan, A. , T. C. Edwards Jr. , and T. Hastie . 2002. “Generalized Linear and Generalized Additive Models in Studies of Species Distributions: Setting the Scene.” Ecological Modelling 157, no. 2–3: 89–100. 10.1016/S0304-3800(02)00204-1.

[ece371800-bib-0025] Hanane, S. , and Z. Amhaouch . 2021. “Spatio‐Temporal Pattern of Latrine Distribution in Reintroduced Cuvier's Gazelles ( *Gazella cuvieri* ): An Assessment in a Mediterranean Forest Reserve.” Biologia 76, no. 11: 3371–3379. 10.1007/s11756-021-00835-5. 34226746 PMC8245920

[ece371800-bib-0026] Hayward, M. , and G. Hayward . 2010. “Potential Amplification of Territorial Advertisement Markings by Black‐Backed Jackals ( *Canis mesomelas* ).” Behaviour 147, no. 8: 979–992.

[ece371800-bib-0027] Hayward, M. W. , and G. I. Kerley . 2009. “Fencing for Conservation: Restriction of Evolutionary Potential or a Riposte to Threatening Processes?” Biological Conservation 142, no. 1: 1–13. 10.1016/j.biocon.2008.09.022.

[ece371800-bib-0028] Hendrichs, H. , and U. Hendrichs . 1971. “Freilanduntersuchungen Zur Ökologie und Ethologie der Zwerg‐Antilope Madoqua (*Rhynchotragus kirki*) (Günther, 1880).” In Dik‐Dik und Elefanten, edited by H. Hendrichs and U. Hendrichs , 9–75. Piper.

[ece371800-bib-0029] IUCN SSC Antelope Specialist Group . 2017. “ *Gazella dorcas* . The IUCN Red List of Threatened Species 2017:E.T8969A50186334.” 10.2305/IUCN.UK.2017-2.RLTS.T8969A50186334.

[ece371800-bib-0030] Jiménez, J. , J. C. Nuñez‐Arjona , C. Rueda , et al. 2017. “Estimating Carnivore Community Structures.” Scientific Reports 7, no. 1: 41036. 10.1038/srep41036. 28120871 PMC5264395

[ece371800-bib-0031] Kacem, S. B. H. , H. P. Müller , and H. Wiesner . 1994. Gestion de la Faune Sauvage et Des Parcs Nationaux en Tunisie: Reintroduction, Gestion et Amenagement, 129–153. Deutsche Gesellschaft für Technische Zusammenarbeit.

[ece371800-bib-0032] Karssene, Y. , M. Chammem , F. Li , A. Eddine , A. Hermann , and S. Nouira . 2019. “Spatial and Temporal Variability in the Distribution, Daily Activity and Diet of Fennec Fox (*Vulpes Zerda*), Red Fox (*Vulpes Vulpes*) and African Golden Wolf (*Caniss Anthus*) in Southern Tunisia.” Mammalian Biology 95: 41–50. 10.1016/j.mambio.2019.02.001.

[ece371800-bib-0071] King, S. R. B. , and J. Gurnell . 2007. “Scent‐making behaviour by stallions: an assessment of function in a reintroduced population of Przewalski horses (*Equus ferus przewalskii*).” Journal of Zoology 272, no. 1: 30–36. 10.1111/j.1469-7998.2006.00243.x.

[ece371800-bib-0033] Legendre, P. , and M. J. Fortin . 1989. “Spatial Pattern and Ecological Analysis.” Vegetatio 80: 107–138. 10.1007/BF00048036.

[ece371800-bib-0034] Louhichi, M. , T. Khorchani , M. Petretto , et al. 2024. “Spatiotemporal Mechanisms of the Coexistence of Reintroduced Scimitar‐Horned Oryx and Native Dorcas Gazelle in Sidi Toui National Park, Tunisia.” Animals 14, no. 10: 1475. 10.3390/ani14101475. 38791692 PMC11117359

[ece371800-bib-0035] Mallon, D. P. , and S. C. Kingswood . 2001. “Antelopes. Part 4: North Africa, the Middle East, and Asia.” In Global Survey and Regional Action Plans. SSC Antelope Specialist Group. IUCN.

[ece371800-bib-0036] Marneweck, C. , A. Jürgens , and A. M. Shrader . 2017a. “Dung Odours Signal Sex, Age, Territorial and Oestrous State in White Rhinos.” Proceedings of the Royal Society B: Biological Sciences 284, no. 1846: 20162376. 10.1098/rspb.2016.2376. PMC5247502 28077775

[ece371800-bib-0037] Marneweck, C. , A. Jürgens , and A. M. Shrader . 2017b. “Temporal Variation of White Rhino Dung Odours.” Journal of Chemical Ecology 43: 955–965. 10.1007/s10886-017-0890-4. 28983753

[ece371800-bib-0038] Martin, L. 2000. “Gazelle (*Gazella* Spp.) Behavioural Ecology: Predicting Animal Behaviour for Prehistoric Environments in South‐West Asia.” Journal of Zoology 250, no. 1: 13–30. 10.1111/j.1469-7998.2000.tb00574.x.

[ece371800-bib-0039] McCullagh, P. 2019. Generalized Linear Models. Routledge. 10.1201/9780203753736.

[ece371800-bib-0040] McGarigal, K. , H. Y. Wan , K. A. Zeller , B. C. Timm , and S. A. Cushman . 2016. “Multi‐Scale Habitat Selection Modeling: A Review and Outlook.” Landscape Ecology 31: 1161–1175. 10.1007/s10980-016-0374-x.

[ece371800-bib-0041] Meliane, M. K. , A. Saidi , M. Petretto , T. Gilbert , and K. Nasri‐Ammar . 2023. “Temporal and Spatial Distribution of Dorcas and Slender‐Horned Gazelles in a Saharan Habitat.” Journal of Wildlife Management 87, no. 5: e22408. 10.1002/jwmg.22408.

[ece371800-bib-0042] Miquelle, D. G. 1991. “Are Moose Mice? The Function of Scent Urination in Moose.” American Naturalist 138, no. 2: 460–477.

[ece371800-bib-0043] Moran, P. A. 1950. “Notes on Continuous Stochastic Phenomena.” Biometrika 37, no. 1/2: 17–23. 10.2307/2332142. 15420245

[ece371800-bib-0044] Muhly, T. B. , C. Semeniuk , A. Massolo , L. Hickman , and M. Musiani . 2011. “Human Activity Helps Prey Win the Predator‐Prey Space Race.” PLoS One 6, no. 3: e17050. 10.1371/journal.pone.0017050. 21399682 PMC3047538

[ece371800-bib-0045] Navarro‐Castilla, A. , B. Sanchez‐Gonzalez , and I. Barja . 2019. “Latrine Behaviour and Faecal Corticosterone Metabolites as Indicators of Habitat‐Related Responses of Wild Rabbits to Predation Risk.” Ecological Indicators 97: 175–182. 10.1016/j.ecolind.2018.10.016.

[ece371800-bib-0046] Ostrowski, S. , and J. B. Williams . 2006. “Heterothermy of Free‐Living Arabian Sand Gazelles (*Gazella Subgutturosa Marica*) in a Desert Environment.” Journal of Experimental Biology 209, no. 8: 1421–1429. 10.1242/jeb.02151. 16574802

[ece371800-bib-0047] Owen‐Smith, N. 1996. “Ecological Guidelines for Waterpoints in Extensive Protected Areas.” South African Journal of Wildlife Research‐24‐Month Delayed Open Access 26, no. 4: 107–112. https://hdl.handle.net/10520/EJC117014.

[ece371800-bib-0048] Pays, O. , P. Blanchard , M. Valeix , et al. 2012. “Detecting Predators and Locating Competitors While Foraging: An Experimental Study of a Medium‐Sized Herbivore in an African Savanna.” Oecologia 169: 419–430. 10.1007/s00442-011-2218-3. 22200851

[ece371800-bib-0049] Pradhan, P. 2016. “Strengthening MaxEnt Modelling Through Screening of Redundant Explanatory Bioclimatic Variables With Variance Inflation Factor Analysis.” Research 8, no. 5: 29–34. 10.7537/marsrsj08051605.

[ece371800-bib-0050] R Core Team . 2023. R: A Language and Environment for Statistical Computing. R Foundation for Statistical Computing.

[ece371800-bib-0051] Ralls, K. , and D. A. Smith . 2004. “Latrine Use by San Joaquin Kit Foxes (*Vulpes Macrotis Mutica*) and Coyotes (*Canis Latrans*).” Western North American Naturalist 64, no. 4: 544–547. https://www.jstor.org/stable/41717412.

[ece371800-bib-0052] Rangel, T. F. , J. A. F. Diniz‐Filho , and L. M. Bini . 2010. “SAM: A Comprehensive Application for Spatial Analysis in Macroecology.” Ecography 33, no. 1: 46–50. 10.1111/j.1600-0587.2009.06299.x.

[ece371800-bib-0053] Roberts, S. C. , and R. I. Dunbar . 2000. “Female Territoriality and the Function of Scent‐Marking in a Monogamous Antelope (*Oreotragus Oreotragus*).” Behavioral Ecology and Sociobiology 47: 417–423. 10.1007/s002650050685.

[ece371800-bib-0054] Roberts, S. C. , and L. M. Gosling . 2004. “Manipulation of Olfactory Signaling and Mate Choice for Conservation Breeding: A Case Study of Harvest Mice.” Conservation Biology 18, no. 2: 548–556. 10.1111/j.1523-1739.2004.00514.x.

[ece371800-bib-0055] Rosell, F. , and L. R. Thomsen . 2006. “Sexual Dimorphism in Territorial Scent Marking by Adult Eurasian Beavers ( *Castor fiber* ).” Journal of Chemical Ecology 32: 1301–1315. 16770720 10.1007/s10886-006-9087-y

[ece371800-bib-0056] Schaller, G. B. 1975. “A Note on a population of *Gazella gazella bennetti* .” Journal of the Bombay Natural History Society 73: 209–211. 10.1007/s10886-006-9087-y.

[ece371800-bib-0057] Soultan, A. , A. Nagy , and O. Attum . 2021. “Midden Site Selection in Dorcas Gazelle: Larger Is Not Always Better.” Ecology and Evolution 11, no. 20: 13661–13667. 34707807 10.1002/ece3.8141 PMC8525146

[ece371800-bib-0058] Sturges, H. A. 1926. “The Choice of a Class Interval.” Journal of the American Statistical Association 21, no. 153: 65–66.

[ece371800-bib-0059] Taha, T. K. , A. M. A. Rashad , A. E. Mahdy , M. A. Aziz , and A. E. Badran . 2018. “Effect of Sex and Age on Body Weight and Some Morphometric Measurements of Gazelle Dorcas and Reedbuck in The Sudan.” Journal of Animal Poultry & Fish Production 7, no. 1: 17–24.

[ece371800-bib-0060] Tarhouni, M. 2008. “Indicateurs de Biodiversité et Dynamique du Couvert Végétal Naturel Aux Voisinages de Trois Points D'eau en Zone Aride Tunisienne: Cas Des Parcours Collectifs d'El‐Ouara.” Thèse de Doctorat en Sciences Biologiques: Faculty of Sciences of Tunis. p. 168.

[ece371800-bib-0061] Tarhouni, M. , W. Ben Hmida , and M. Neffati . 2014. “Caractérisation du Couvert Végétal Naturel à L'extérieur et à L'intérieur du Parc National de Sidi Toui, Zone Aride de la Tunisie.” Ecologia Mediterranea 40, no. 2: 41–52.

[ece371800-bib-0062] Tucker, M. A. , K. Böhning‐Gaese , W. F. Fagan , et al. 2018. “Moving in the Anthropocene: Global Reductions in Terrestrial Mammalian Movements.” Science 359, no. 6374: 466–469. 10.1126/science.aam9712. 29371471

[ece371800-bib-0063] Vanak, A. T. , M. Thaker , and R. Slotow . 2010. “Do Fences Create an Edge‐Effect on the Movement Patterns of a Highly Mobile Mega‐Herbivore?” Biological Conservation 143, no. 11: 2631–2637. 10.1016/j.biocon.2010.07.005.

[ece371800-bib-0064] Walther, F. R. 1979. Verhalten Von Gazellen. Ziemsen Verlag.

[ece371800-bib-0065] Walther, F. R. , E. C. Mungall , and G. A. Grau . 1983. Gazelles and Their Relatives: A Study in Territorial Behavior. Noyes.

[ece371800-bib-0066] Whittingham, M. J. , P. A. Stephens , R. B. Bradbury , and R. P. Freckleton . 2006. “Why Do We Still Use Stepwise Modelling in Ecology and Behaviour?” Journal of Animal Ecology 75, no. 5: 1182–1189. 10.1111/j.1365-2656.2006.01141.x. 16922854

[ece371800-bib-0067] Woodroffe, R. , S. Hedges , and S. M. Durant . 2014. “To Fence or Not to Fence.” Science 344, no. 6179: 46–48. 10.1126/science.1246251. 24700847

[ece371800-bib-0068] Wronski, T. , A. Apio , M. Plath , and M. Ziege . 2013. “Sex Difference in the Communicatory Significance of Localized Defecation Sites in Arabian Gazelles ( *Gazella arabica* ).” Journal of Ethology 31: 129–140. 10.1007/s10164-012-0357-6.

[ece371800-bib-0069] Wronski, T. , and M. Plath . 2010. “Characterization of the Spatial Distribution of Latrines in Reintroduced Mountain Gazelles: Do Latrines Demarcate Female Group Home Ranges?” Journal of Zoology 280, no. 1: 92–101. 10.1111/j.1469-7998.2009.00643.x.

[ece371800-bib-0070] Zoromski, L. D. , R. W. DeYoung , J. A. Goolsby , et al. 2022. “Latrine Ecology of Nilgai Antelope.” Journal of Mammalogy 103, no. 5: 1194–1207. 10.1093/jmammal/gyac056.

